# COVID-19 Vaccination in Pregnancy: Pilot Study for Maternal and Neonatal MicroRNA Profiles

**DOI:** 10.3390/vaccines11121814

**Published:** 2023-12-04

**Authors:** Wei-Chun Chen, Shu-Yu Hu, Ching-Fen Shen, Mei-Hsiu Cheng, Jun-Jie Hong, Ching-Ju Shen, Chao-Min Cheng

**Affiliations:** 1 Institute of Biomedical Engineering, National Tsing Hua University, Hsinchu 300, Taiwan; lionsmanic@gmail.com (W.-C.C.); chloe.natshuun@gmail.com (S.-Y.H.); 2 Division of Gynecologic Oncology, Department of Obstetrics and Gynecology, Chang Gung Memorial Hospital at Linkou, College of Medicine, Chang Gung University, Taoyuan 333, Taiwan; 3 Department of Obstetrics and Gynecology, New Taipei City Municipal Tucheng Hospital, New Taipei City 236, Taiwan; 4 International Intercollegiate Ph.D. Program, National Tsing Hua University, Hsinchu 300, Taiwan; 5 School of Traditional Chinese Medicine, Chang Gung University, Taoyuan 333, Taiwan; 6 Department of Pediatrics, National Cheng Kung University Hospital, College of Medicine, National Cheng Kung University, Tainan 701, Taiwan; drshen1112@gmail.com; 7 Taiwan Business Development Department, Inti Taiwan, Inc., Hsinchu 302, Taiwan; michelle@intilabs.com (M.-H.C.); winterhong@intilabs.com (J.-J.H.); 8 Department of Obstetrics and Gynecology, Kaohsiung Medical University Hospital, Kaohsiung Medical University, Kaohsiung 807, Taiwan

**Keywords:** COVID-19 vaccines, SARS-CoV-2, miRNA, pregnant, neonatal, cord blood, Pfizer BioNTech, Moderna

## Abstract

This pilot study explores alterations in miRNA profiles among pregnant women and their neonates upon receiving different doses of COVID-19 vaccines. Blood samples, including maternal blood (MB) and neonatal cord blood (CB), collected from five pregnant women were scrutinized using the miRNA PanelChip Analysis System, identifying nine distinct miRNAs, including miR-451a and miR-1972, which exhibited significant downregulation with two vaccine doses in both MB and CB. When compared with women vaccinated with four doses, miR-486-5p, miR-451a, and miR-1972 in the two-dose group also showed notable downregulation. Evaluating recipients of three and four doses, miR-423-5p and miR-1972 expression were significantly reduced in both MB and CB. Further comparative analysis highlighted a decline in miR-223-3p expression with increasing vaccine doses, while miR15a-5p, miR-16-5p, and miR-423-5p showed an upward trend. Notably, miR-451a, miR-1972, and miR-423-5p levels varied across doses and were associated with pathways such as “PI3K-Akt”, “neurotrophin signaling”, and “cortisol synthesis”, suggesting the profound influence of vaccination on diverse molecular mechanisms. Our research has uncovered that escalating vaccine dosages impact miRNA profiles, which may be associated with the immunological response mechanisms in both the mother and fetus, thus indicating a substantial impact of vaccination on various molecular processes.

## 1. Introduction

Since 2019, the global community has faced the severe ramifications of the COVID-19 (Coronavirus Disease 2019) pandemic, induced by the highly contagious SARS-CoV-2 virus [1]. This health emergency revealed an impressive transmission rate and placed immense burdens on healthcare infrastructures and the global economy. In response to this crisis, the development and widespread promotion of vaccines have become pivotal [2]. These vaccines significantly mitigate the virus’s spread and notably reduce severe case incidences, particularly among high-risk demographics, including healthcare professionals, the elderly, and pregnant women. Various studies underscore the enhanced susceptibility of pregnant women to the virus, indicating a rise in complications such as preeclampsia and preterm births [3,4,5]. Amid these difficulties, the successful development and deployment of COVID-19 vaccines stand as a beacon of scientific achievement and human tenacity.

COVID-19 vaccinations assist expectant mothers in generating and passing on vital protective antibodies to their fetuses through a mechanism known as transplacental transmission [6,7,8,9]. This process, which prominently involves the transfer of SARS-CoV-2 neutralizing antibodies (Nabs), promises potential protection for both fetuses and newborns [6,7,8,9]. Intriguingly, this protective mantle seems to extend its coverage even to emerging SARS-CoV-2 strains, including the Omicron variant [10]. Our previous research, along with corroborating studies, consistently highlights the criticality of these Nab transfers [8,10,11]. Such defenses furnish newborns with a layer of immunity against pernicious threats like SARS-CoV-2, further endorsing the vaccination of pregnant women [12,13]. However, the introduction of the Omicron variant and its vaccine-resistant subvariants instigated grave concerns. This, in turn, catalyzed the evolution of bivalent boosters, designed to enhance the body’s defensive response [14]. The enhancement in protective efficacy becomes increasingly evident with the administration of multiple vaccine doses, especially when utilizing bivalent COVID-19 vaccines [10].

The miRNAs are involved not only in the cellular antiviral response but also in regulating the replication and spread of viruses [15]. For SARS-CoV-2, the mechanism of infection might be mediated by the virus’s own miRNAs interacting with host functional genes and their regulatory areas. This affects processes like viral replication, membrane fusion, and penetration into host cells [16]. Interestingly, human cells can produce their own miRNAs to counteract SARS-CoV-2 infections [17]. Distinct miRNA profiles were observed between general ward and ICU COVID-19 patients. For instance, miR-27a-3p, miR-27b-3p, miR-148a-3p, miR-199a-5p, and miR-491-5p expressions were heightened in ICU patients [18]. Plasma miR-1-3p, miR-126-3p, and miR-146a-5p levels stand out as predictors for the severity and outcome of COVID-19-related respiratory distress, showing significant elevation in severe cases [19]. Moreover, a combination of miR-1246, miR-4433b-5p, miR-4467, and miR-619-5p in plasma offers a potential biomarker to distinguish between COVID-19 patients and healthy subjects [20].

Certain drugs and interventions can potentially modulate the course of infection by regulating miRNA. For instance, the intake of polyphenols has been reported to mitigate the symptoms of several chronic ailments, including type 2 diabetes, cardiovascular disease, and notably, COVID-19 [21]. Intriguingly, vaccinations themselves induce alterations in the body’s miRNA profile, and these changes could subsequently influence the physiologically prophylactic response to COVID-19. Notably, there is a conspicuous absence of literature investigating miRNA profiles and their variations in maternal peripheral blood and neonatal cord blood post vaccination. Thus, the primary objective of this pilot study is to explore the alterations in miRNA profiles in mothers and their neonates following the administration of varying doses of COVID-19 vaccines. We anticipate that this investigation will provide deeper insights into the mechanistic action of COVID-19 vaccines and their potential ramifications.

## 2. Materials and Methods

### 2.1. Study Design and Patient Selection

We conducted this prospective study at Kaohsiung Medical University Hospital. All participants were identified as singleton pregnancies and enrolled in the current study during hospitalization for baby delivery. All of the participants were required to have received 2 to 4 doses of mRNA-based COVID-19 vaccines including the Pfizer BioNTech (BNT162b2) COVID-19 vaccine or the Spikevax (elasomeran) COVID-19 vaccine (previously called the mRNA-1273 Moderna vaccine). Those participants receiving a 4th dose received the Moderna COVID-19 bivalent (SPIKEVAX Bivalent Original/Omicron BA.1) vaccine. Additionally, the last dose of COVID-10 vaccination was required to be administered during pregnancy. In our study, participants undergoing antenatal care were permitted to receive other routine vaccinations. These included the tetanus toxoid, reduced diphtheria toxoid, and acellular pertussis (Tdap) vaccines (Adacel, Sanofi Pasteur, Toronto, ON, Canada) and the influenza (Flu) vaccine (AdimFlu-S, QIS, Adimmune Corporation, Taichung, Taiwan; FlucelvaxQuad, CSL Behring GmbH, Marburg, Germany; ad VAXIGRIP TETRA, Sanofi Pasteur, Val-de-Reuil, Cedex, France). No obvious discomfort was recorded after participants received any of the above vaccinations.

The inclusion and exclusion criteria in our study were as follows: (1) age threshold of 20 or above; (2) no cases of early labor; (3) no previous or current history of chronic illnesses requiring immunosuppressants; (4) no history of cancer that demanded specific treatments; (5) no pregnancy-related illness such as gestational hypertension or diabetes; and (6) no previous history of COVID-19 disease and SARS-CoV-2 infection. Additionally, qualified participants were enrolled after signing the informed consent form, and the study was performed following approval by the local institutional review board (IRB) (IRB number: KMUHIRB-SV(II)-20210087).

### 2.2. Specimen and Clinical Information Gathering and Sampling

On the day of childbirth, maternal peripheral blood and neonatal umbilical cord blood (after clamping) were collected. These samples were immediately sent for specialized laboratory analysis. Additionally, we also accessed and recorded associated clinical metrics from electronic medical records. We explored various maternal attributes, such as age, body mass index (BMI), prior pregnancies, and gestational weeks. Neonatal factors such as birth weight and gender were also included. Clinical vaccination data including doses of COVID-19 vaccines, the dates and types of COVID-19 vaccines received, and intervals between doses were also collected. All the aforementioned information was compiled for subsequent investigation.

### 2.3. Neutralizing Antibody Inhibition Rate of SARS-CoV-2 Omicron BA.5

We utilized the ELISA kit (ACROBiosystems Cat. No. RAS-N107) to essay the neutralizing antibody inhibition rate against SARS-CoV-2 Spike RBD of the samples. The human ACE2 protein was pre-coated on the plate; we then added the samples and controls into the well followed by the addition of HRP-SARS-CoV-2 Spike RBD. After incubation in the dark at 37 °C for an hour, we washed away the unbounded samples. Subsequently, the substrate solution was added to the well, and incubated in the dark at 37 °C for 20 min. Finally, we added a stop solution and read the absorbance at 450 nm/630 nm using an ELISA reader.

### 2.4. Extraction, Subsequent Reverse Transcription, and the Detection of miRNAs

Maternal and neonatal umbilical cord plasma were obtained via centrifugation and miRNA was extracted using the miRNeasy Serum/Plasma Advanced Kit (Cat. No. 217204, Qiagen, Hilden, Germany) following the manufacturer’s guidelines. The isolated miRNAs were then eluted in 20 μL of nuclease-free water and subsequent quantification of the extracted miRNA concentration was carried out using the Qubit™ microRNA Assay Kit (Cat. No. Q32880, Thermo Fisher Scientific, Waltham, MA, USA). We subsequently proceeded to the synthesis of cDNA. The above reverse transcription procedure was carried out using QuarkBio’s microRNA Reverse Transcription kit (Quark Biosciences, Inc., Hsinchu, Taiwan) according to the manufacturer’s instructions.

The miRNA was subsequently identified using the MIRAscan assay and the NextAmp™ Analysis System. MIRAscan (Inti Taiwan, Inc., Hsinchu, Taiwan) works in tandem with the NextAmp™ System (Quark Biosciences, Inc., Hsinchu, Taiwan), which employs real-time quantitative PCR for the measurement of gene expression, leveraging the PanelChip^®^ technology integral to the NextAmp™ System. The PanelChip^®^ is a compact chip measuring 36 mm × 36 mm × 1 mm. Each chip contains 2500 nanowells, each of which is capable of conducting a single real-time PCR reaction. This allows for the simultaneous testing of multiple genes on a single chip using the PanelChip^®^ technology. In the real-time quantitative PCR process, 0.15 ng of cDNA was combined with QuarkBio’s qPCR master mix. Thermal cycling for qPCR was executed using the Q Station™ (Quark Biosciences, Inc., Hsinchu, Taiwan). The cycle consisted of an initial 36-s phase at 95 °C, followed by 40 rounds of 72 s at 60 °C. This MIRAscan assay is designed to detect as many as 83 distinct miRNAs, which can be used to gauge disease status and various physiological conditions.

### 2.5. Statistics: The Identification and Further Analysis of miRNAs

Samples were subjected to a multi-gene qPCR reaction using the MIRAscan PanelChip^®^ platform as described above. Raw cycle quantification (Cq) values representing miRNA concentrations were generated for subsequent computational analyses. Post acquisition, a data-cleaning phase was initiated to remove miRNAs lacking amplification signals, and normalization was performed using an internal control. Normalized miRNA expression levels were denoted as ∆Cq values obtained by subtracting control values. We further employed clustering analysis to assess variations in microRNA expression levels in both maternal and neonatal blood samples taken from participants receiving different doses of COVID-19 vaccines. The clustering analysis was guided by predetermined thresholds for fold change and *p*-values. From the variation patterns of the miRNAs with significant differential expression in the sample, we could further identify outlier samples that might interfere with the general results and thus determine the most representative miRNAs for our research. Differential expression of miRNAs between the two groups was determined based on relative changes in Cq values, labeled as ∆∆Cq. Any miRNA exhibiting a significant expression difference was identified using the criterion “|∆∆Cq| ≥ log_2_1.5 ≈ 0.585”, and it indicates at least a 1.5-fold change in gene expression, which is commonly considered significant.

For further scrutiny of the miRNAs with substantial differential expression, MicroRNA Target Interaction (MTI) analysis was performed using the miRTarBase—a comprehensive database featuring experimentally validated and evidence-based miRNA interactions gathered from a multitude of scholarly publications. MTIs backed by robust experimental evidence, such as qPCR, reporter assays, and Western blotting, were selected for additional assessments. These chosen MTIs were then employed in gene set enrichment and functional analyses via the clusterProfiler tool.

By using this analytical platform, we were able to identify specific miRNAs that exhibited significant expression differences between samples obtained from pregnant women and their newborns, following receipt of varying pre-childbirth doses of COVID-19 vaccines. These findings offer valuable insights into how different dosages of COVID-19 vaccines might influence miRNA expression profiles.

## 3. Results

### 3.1. Participant Data

A total of five participants were enrolled in our study, as presented in Table 1. The age range among the participants was 27 to 38 years. Among the participants, two were experiencing their first pregnancy, while the remaining three were in their second pregnancy. The body mass index (BMI) of our cohorts ranged from 23 to 28. All participants had a full-term pregnancy, with childbirth at gestational ages ranging between 38 to 40 weeks. With regard to COVID-19 vaccination, two participants had received two doses, another two had received three doses, and one had received four doses. For those who received either two or three doses, the mRNA-1273 Moderna vaccine was administered. However, for the participants who received four doses, the first three were BNT162b2 vaccines and the fourth dose was a SPIKEVAX Bivalent BA.1 vaccine. Notably, the final dose of COVID-19 vaccine for each participant was administered during pregnancy. As for the newborns, their weights ranged from 2500 to 3200 g. Only one of the infants was female, while the rest were male. Additionally, three of the participants had been vaccinated with Tdap and Flu vaccines; these were the same individuals who had received two, three, or four doses of COVID-19 vaccines. The remaining two participants had not received either the Tdap or Flu vaccination.

We further evaluated the neutralizing antibody (Nab) inhibition rate against the Omicron BA.5 subvariant of SARS-CoV-2 for each participant in Table S1. Due to insufficient sample amount, data for maternal blood in cases 1 and 2 who received two doses, and neonatal cord blood in the case of four doses, were unfortunately lacking. Nevertheless, it is observable that the Nab inhibition rate in the maternal blood of participants with four doses was significantly higher compared with the average of two participants with three doses (85.31% vs. 24.94%). Similarly, in the neonatal cord blood, the average Nab inhibition rate for three doses was higher than that for two doses (14.61 vs. 7.15%). This finding indicates an increase in Nab inhibition rate with the administration of a greater number of vaccine doses.

### 3.2. miRNA Expression Data and Subsequent Data Extraction

**Figure 1:**
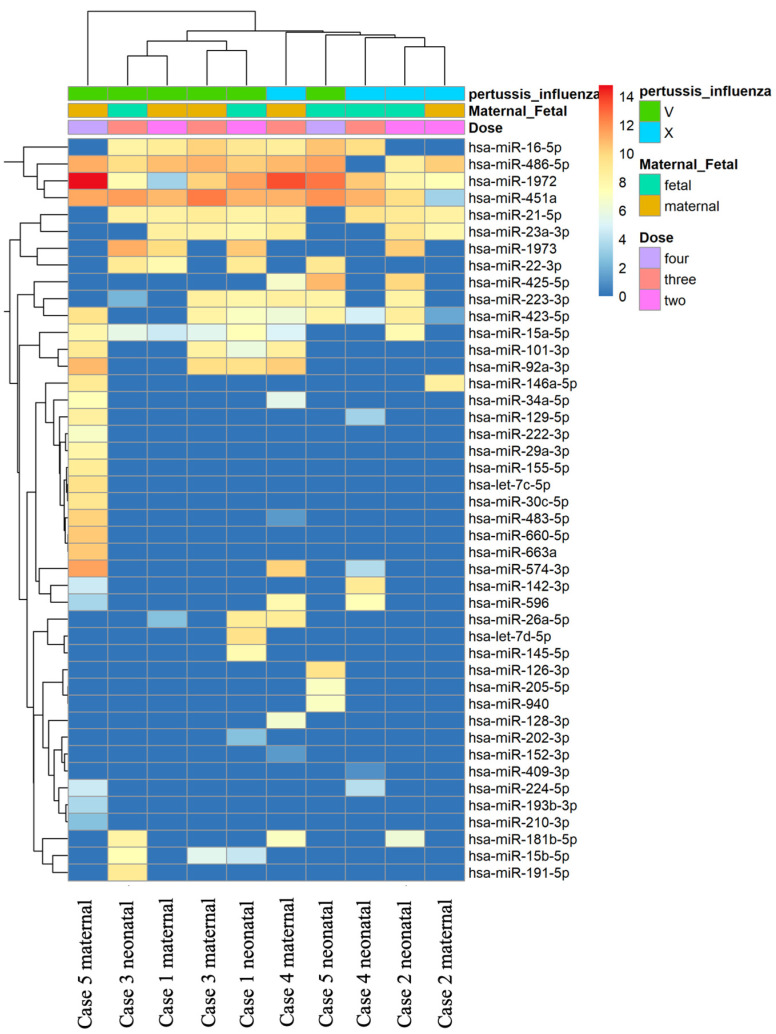
Heatmap of all miRNA data in all samples. The heatmap was generated using the miRNA PanelChip Analysis System and created post exclusion of miRNAs that were not expressed in any of the samples. Within the heatmap, hues trending towards red signify an enhancement in miRNA expression, whereas those leaning towards blue indicate a lack or absence of miRNA expression. Despite the identification of a multitude of miRNAs, no discernible group trends were observed across all samples.

**Figure 2:**
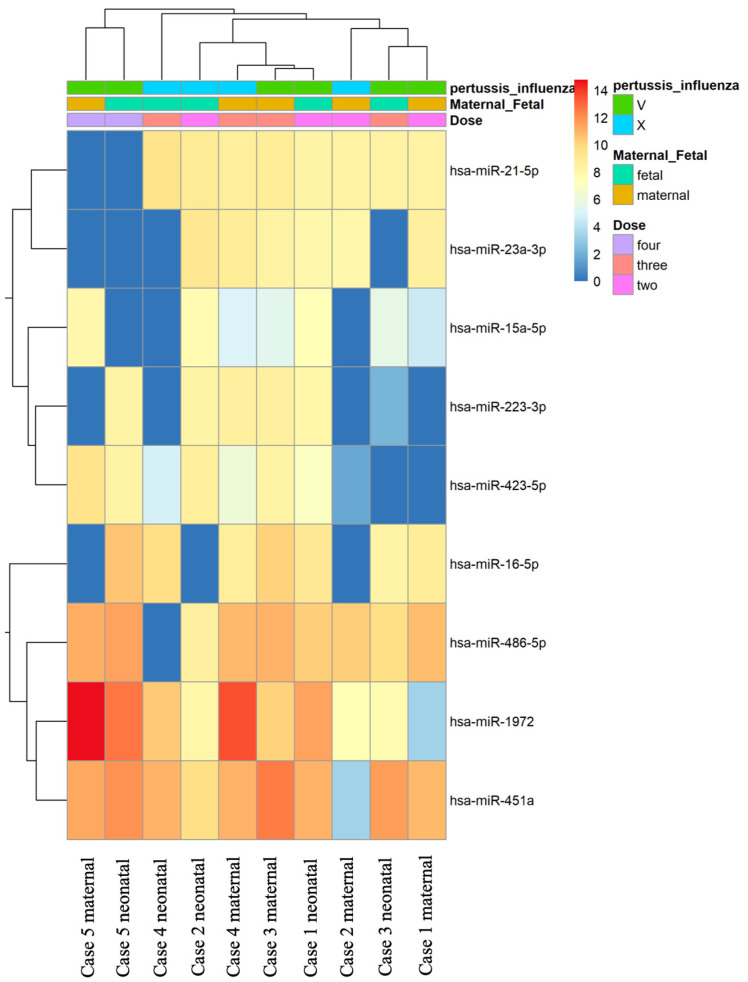
Heatmap of filter data of the miRNAs in the samples. Further refinement from Figure 1 involved the exclusion of miRNAs unexpressed in more than five samples. This process led to the selection of nine miRNAs for comparative analysis, including miR-21-5p, miR-23a-3p, miR-15a-5p, miR-223-3p, miR-423-5p, miR-16-5p, miR-486-5p, miR-1972, and miR-451a. The resulting heatmap of these filtered miRNAs reveals that samples with four vaccine doses form a distinct cluster.

In our study, the miRNA PanelChip was used to detect miRNA expressions from the collected samples. The resulting data were used to create the heatmap displayed in Figure 1. It should be noted that this heatmap was generated after the exclusion of miRNAs without expression in any of the specimens. Within the heatmap, colors closer to red indicate enhanced miRNA expression, while those closer to blue denote a lack of miRNA expression. Despite the multitude of miRNAs identified, no discernible group trends could be observed among all samples. To gain a better understanding of the relationships between the samples, further filtering was conducted. We retained miRNAs that were expressed in the majority of samples, eliminating any miRNAs that were unexpressed in more than five samples. This led to the generation of Figure 2, which presents a heatmap of the filtered data. A total of nine miRNAs were selected for our comparative analysis, including miR-21-5p, miR-23a-3p, miR-15a-5p, miR-223-3p, miR-423-5p, miR-16-5p, miR-486-5p, miR-1972, and miR-451a.

Subsequent clustering analysis was performed using Principal Component Analysis (PCA), as shown in Figure S1. Based on the first two principal components (PC1 and PC2), four distinct quadrants were identified, allowing for differentiation by dosage groups. Notably, mothers and fetuses who received four doses of the vaccine could be independently categorized. The sample labeled “two_fetal_396” from case 1 was identified as an outlier and was, therefore, excluded from further analysis.

### 3.3. miRNA with Significant Differential Expression Simultaneously in Maternal Blood and Cord Blood between Different Vaccination Doses

In our subsequent analysis, we focused on miRNAs with significantly differential expression in maternal peripheral blood and neonatal umbilical cord blood among pregnant women who received different doses of COVID-19 vaccines. We identified the intersecting miRNAs with differential expression in both maternal and neonatal blood, as listed in Table 2. Our analysis revealed that pregnant women who received two doses of COVID-19 vaccines, along with their newborns, presented significantly differential expression of two miRNAs—miR-451a and miR-1972—compared with those who received three doses. These findings are illustrated in Figure S2 and indicate a significant downregulation of the two miRNAs in the two-dose group.

A comparison between individuals who received two doses and those who received four doses of COVID-19 vaccines is depicted in Figure S3. Three miRNAs were found to exhibit significant differential expression in both maternal and neonatal blood. These include miR-451a and miR-1972, which were also observed in the comparison between the two- and three-dose groups, as well as an additional miRNA, miR-486-5p. All three miRNAs showed a notable decrease in expression in the two-dose vaccination group. Further comparison between the three-dose and four-dose vaccination groups is illustrated in Figure S4. Two miRNAs—miR-423-5p and miR-1972—showed significant differential expression in both maternal and neonatal blood. Compared with the participants with four vaccine doses, these two miRNAs exhibited significantly reduced expression in the three-dose group. It is worth noting that miR-1972 was consistently identified in both the two-dose/three-dose and two-dose/four-dose comparisons.

### 3.4. miRNA with Significant Differential Expression Simultaneously in Maternal Blood and Neonatal Cord Blood between “2 Dose and 3 Dose” as Well as “2 Dose and 4 Dose” Groups

In our study, we employed different methods to investigate the differentially expressed microRNAs (miRNAs) in maternal blood and neonatal cord blood samples after COVID-19 vaccination. In addition to the intersectional analysis of miRNA levels in maternal and fetal blood, we utilized an alternative approach to identify miRNAs with significantly differential expression. We calculated the average miRNA levels in maternal and umbilical cord blood samples and then compared these averages between two groups: those who received two or three doses and those who received two or four doses of COVID-19 vaccines. The ΔΔCq values were obtained and differences with an absolute value ≧ 1 were considered significant. The miRNAs that displayed meaningful trends are listed in Table 3.

Our analysis revealed that certain miRNAs exhibited dose-dependent trends in expression levels. For instance, miR-223-3p demonstrated a decrease in expression as the number of vaccine doses increased. On the other hand, miR15a-5p, miR-16-5p, and miR-423-5p showed an overall increasing trend in expression levels with increasing vaccine doses. Notably, the expression level of miR15a-5p initially decreased from two to three doses but increased more substantially from two to four doses, confirming its overall increasing trend. This approach allows for a nuanced understanding of the miRNA profiles, and notably, provides insights that are particularly useful for indirectly observing the differences between receiving three and four doses of the vaccine.

### 3.5. Functional Pathway of miRNA with Significant Differential Expression

In our investigation of miRNA with significantly differential expression profiles observed in maternal and umbilical cord blood samples following different doses of COVID-19 vaccines, we identified functional pathways with significance, as detailed in Table 4 and Table S2–S5. Since numerous pathways can be detected, we presented the pathways related to immune or inflammation mechanisms, and excluded pathways associated with cancer or disease. We also provided *p*-values to signify the statistical relevance of each functional pathway. If more than ten pathways exhibited significance, only the top ten were listed. Additionally, the corresponding genes involved in these pathways were enumerated.

Notably, several miRNAs, including miR-451a, miR-1972, and miR-423-5p, were consistently present in samples across different vaccination dosages. The functional pathways associated with miR-451a were most prominently linked to “PI3K-Akt” and “estrogen signaling pathways”. Meanwhile, six significant functional pathways were associated with miR-1972, with “RNA polymerase” and “neurotrophin signaling pathways” showing the strongest correlation. For miR-423-5p, the most significant pathways were “cortisol synthesis and secretion” and “parathyroid hormone synthesis, secretion, and action”. Additionally, “cellular senescence” and “p53 signaling pathway” were most significant for miR-486-5p. For miR-16-5p, the prominent pathways included “PI3K-Akt signaling”, “mTOR signaling”, “cell cycle”, and “EGFR tyrosine kinase inhibitor resistance”, while miR-15a-5p significantly involved “PI3K-Akt signaling pathway”, “cell cycle”, “p53 signaling pathway”, and “signaling pathways regulating pluripotency of stem cells”. Similarly, miR-223-3p showed significant involvement in pathways like cellular senescence, FoxO signaling, p53 signaling, cell cycle, and endocrine resistance.

It is evident that many of these miRNAs share common pathways, such as PI3K-Akt signaling, cellular senescence, p53 signaling, and cell cycle. This suggests that the variation in vaccination dosages impacts miRNA expression, which, in turn, influences the functional pathways related to the different vaccine doses administered.

## 4. Discussion

In this study, we sought to elucidate the physiological variations in maternal and umbilical cord blood among pregnant women who received different doses of COVID-19 vaccines by examining the differential expressions of miRNA under varying physiological conditions. Our findings indicate that when comparing the two-dose and three-dose groups, miR-451a and miR-1972 showed decreased expression levels in the two-dose group in both maternal and umbilical cord blood samples. A similar trend was observed between the two-dose and four-dose groups, with miR-451a, miR-1972, and miR-486-5p all exhibiting lower expressions in the two-dose group. Comparatively, in the context of the three-dose versus four-dose groups, miR-423-5p and miR-1972 demonstrated decreased expression in the three-dose group. On average, only miR-223-3p displayed a reduction in expression as the number of vaccine doses increased. Conversely, miR-15a-5p, miR-16-5p, and miR-423-5p all exhibited increased expression as the number of doses escalated.

The molecular mechanisms underlying SARS-CoV-2 infection involve the virus’s own miRNAs interacting with functional genes and their regulatory regions, influencing processes such as viral replication, membrane fusion, and cell entry [16]. Notably, viral miRNAs can bind to host miRNAs during the infection, targeting immunity-associated genes and affecting signaling pathways of tumor necrosis factor (TNF) and chemokines [22]. For instance, MR66-3p influences TNF-α, while MR198-3p impacts IFN [16]. Human host cells can express their own miRNAs as a defense against SARS-CoV-2. MiRNAs like miR-125a, miR-141, and hsa-miR-200 have been observed to inhibit ACE2 mRNA [23,24]. In contrast, miR-497-5p, hsa-miR-21-3p, and hsa-miR-195-5p can degrade the RNA of SARS-CoV-2’s coding regions, thereby suppressing viral replication [25]. Additionally, miRNA-323 and miRNA-485 target ORF1a/b, a gene-encoding enzyme essential for SARS-CoV-2 replication and translation [26]. MiRNAs present a promising avenue for diagnosing COVID-19 infection and predicting post-infection outcomes. For confirmed COVID-19 cases, blood samples have shown differential miRNA expression: downregulation of miR-17-5p and miR-142-5p and upregulation of miR-15a-5p, miR-19a-3p, miR-19b-3p, miR-23a-3p, miR-92a-3p, and miR-320a [27]. Clear distinctions in miRNA expression, such as MiR-155, miR-208a, and miR-499, have been identified between COVID-19 and flu-induced ARDS patients [28]. Compared with ward patients, ICU patients demonstrate increased expression of miR-27a-3p, miR-27b-3p, miR-148a-3p, miR-199a-5p, and miR-491-5p [18]. A combination of miR-1246, miR-4433b-5p, miR-4467, and miR-619-5p can differentiate between COVID-19 patients and healthy individuals [20]. Hence, some therapeutic strategies may harness the regulatory potential of miRNAs for controlling infections. Polyphenol intake, for example, has been observed to alleviate symptoms of various chronic diseases, including type 2 diabetes, cardiovascular disease, and even COVID-19 [21]. Vaccination itself triggers changes in miRNA expression within the body, potentially influencing the body’s protective response to COVID-19. Previous research has indicated that post vaccination, there might be changes in the expression of miR-192, affecting IL-6 levels [29]. MiR-21’s expression also alters post vaccination, regulating the expression of IL-12 necessary for Th1 responses. MiR-451a can impact both IFN and IL-6 expression, which may influence vaccine efficacy and vaccine-induced inflammation [29]. Notably, levels of miR-92a-2-5p are inversely correlated with vaccine-adverse reactions, while EV miR-148a levels directly correlate with vaccine antibody concentrations [30]. In pregnant women, no prior studies have been conducted. Our paper confirms the aforementioned distinctions in this population.

In our study, we observed that with each additional dose of COVID-19 vaccine administered to pregnant women, there was a progressive increase in the expression of miR-451a and miR-1972, especially between the second and third doses and the third and fourth doses. Previous research has shown that in severely affected patients, there is a notable decrease in the levels of miR-451a (fold change = 0.58) [18]. Additionally, dysregulation of miR-451a correlates with lymphocyte and neutrophil counts, as well as concentrations of D-dimer, ferritin, and CRP. These miRNAs, showing significant alterations, play a role in modulating various aspects of immune and inflammatory pathways, such as the synthesis of cytokines and chemokines, such as miR-451a [18]. Analyses of hospitalized severe COVID-19 patients in other studies have also identified IL-6, IL-10, CCL20, and miR-451a as key factors closely related to COVID-19 mortality [31]. Past research has pinpointed three upregulated lncRNAs—LOC105371414, LOC105374981, and LOC107987081—that carry binding sites for miR-451a and might compete with IL-6R for miR-451a binding [32]. In healthy donors, miR-451a helps maintain normal levels of IL-6R/CCL2 by targeting IL-6R/CCL2 mRNAs. However, in COVID-19 patients, a decrease in miR-451a expression coupled with its binding to lncRNAs could amplify IL-6R/CCL2 expression at the protein level [32]. Consequently, reduced miR-451a and increased lncRNA may intensify the cytokine storm induced by IL-6 in COVID-19 patients. Our findings further indicate that as the number of vaccine doses rises, so does the expression of miR-451a, potentially aiding in maintaining normal levels of IL-6R.

In previous studies, miR-1972 has been identified to regulate various targets, including EDN1, CD274, and PDCD1LG2 [33]. These targets play pivotal roles in processes such as respiratory burst and T-cell activation, highlighting miR-1972’s association with T and B cell signaling as well as TNFR1 signaling pathways [34]. Notably, in the context of chronic myeloid leukemia, overexpression of miR-1972 has been linked to cell cycle arrest at the G2-M phase [35]. Additionally, in ovarian cancer, miR-1972 serves an oncogenic role, where its elevated expression correlates with cisplatin-resistant ovarian cancers. Intriguingly, inhibiting miR-1972 has been shown to curb the proliferation of these cisplatin-resistant ovarian cancer cells, thereby enhancing their sensitivity to cisplatin [36]. However, the role of miR-1972 following COVID-19 vaccine administration in pregnant women remains elusive. Our research indicates that with increasing vaccine doses, there is a subsequent rise in the expression of miR-1972, suggesting its potential involvement in immunological mechanisms related to T-cell signaling.

In our study, distinct variations in the expression levels of specific miRNAs were observed across varying doses in maternal and umbilical cord blood. Specifically, miR-486-5p levels differed between doses two and four, while miR-423-5p showed differences between doses three and four. In both cases, the expression increased with a higher number of doses. Levels of miR-486-5p, highly expressed in the hematopoietic system, particularly within erythrocytes, also tend to be released during hemolysis, complicating its interpretation in biomarker studies due to increased levels in hemolyzed samples [37,38,39]. This miRNA is among the most abundant in exosomes derived from human adipose and bone marrow stromal cells (BMSCs) and has demonstrated protective effects in organ injury experimental models [40]. Differential expression of miR-486-5p in human plasma or serum is evident in several conditions, including sepsis, cardiopulmonary diseases, osteoarthritis, endocrine disorders, and type 2 diabetes [41,42,43]. Notably, a decrease in miR-486-5p levels was identified in critically ill COVID-19 patients, correlating with neutrophil and lymphocyte counts, as well as D-dimer and ferritin concentrations [18]. Further, miR-486-5p plays a role in antiviral mechanisms against influenza A viruses when expressed in respiratory epithelial cells and induces inflammation in acute lung injury by targeting OTUD7B [44,45]. However, other research also reported that overexpression was noted in severe COVID-19 cases [46]. Intriguingly, an increase in miR-486-5p has been observed in pregnant women receiving varying vaccine doses. Although the current role is unclear, it may also be related to immune modulation associated with protecting against organ damage.

In recent studies, miR-423-5p has been observed to exhibit increased expression following SARS-CoV-2 infection. This microRNA can regulate the expression of MALAT1 and has been found to impede MALTA1-mediated proliferation, tumor growth, and metastasis [47]. Historically, miR-423-5p has been associated with the modulation of various tumor developments. Specifically, it intensifies the progression of lung adenocarcinoma by targeting CADM1, promotes the progression of prostate cancer by targeting GRIM-19, and suppresses the proliferation and invasion of osteosarcoma by targeting STMN1 [48,49]. A decrease in miR-423-5p expression was linked to a reduction in the proliferation and tumorigenic capacities of HCC cells, simultaneously facilitating apoptosis within these cells [50]. This suggests a pivotal role in the regulation of immune escape in HCC mediated by Tregs [50]. Elevated circulating expressions of miR-423-5p have been detected during heart failure and pulmonary tuberculosis [51,52]. Furthermore, a combination measurement of three miRNAs, namely miR-423-5p, miR-23a-3p, and miR-195-5p, has been demonstrated to identify early-stage COVID-19 with an impressive accuracy of 99.9% [53]. The upregulated expression of miR-423-5p post-SARS-CoV-2 infection might contribute to the host’s defense mechanisms by aiding in the clearance of infected cells. While there have been no prior reports on the expression of miR-423-5p in pregnant women receiving varying doses of COVID-19 vaccines, it is evident that with increased vaccinations, there is an uptick in expression levels. This might also be related to the potential ability of miR-423-5p to participate in clearing infected cells within the body.

Upon averaging values from maternal blood and umbilical cord blood, it was observed that the expression of miR-15a-5p and miR-16-5p increased concomitantly with increasing doses. In contrast, the expression of miR-223-3p decreased with a rise in dosage. Haddad et al. identified that miR-16-5p and miR-15a-5p can bind to the single-stranded RNA of the full-length SARS-CoV-2 genome [25]. Similarly, Fayyad-Kazan et al. highlighted a differential expression of miR-15a-5p between patients with SARS-CoV-2 and healthy controls [27]. Previous research findings indicate that the SARS-CoV-2 RNA hinders the binding between AGO/miR-15a-5p and its mRNA targets, leading to their decreased expression [54]. It is noteworthy that miR-15a-5p is implicated in the modulation of the PD-1–PD-L1 interaction, aiding in the restoration of T-cell immunity to counteract malignant tumors [55]. Furthermore, miR-15a-5p might be involved in the inflammatory cascade during sepsis, potentially via the activation of the NF-κB pathway and targeting TNIP2 [56]. Overexpression of miR-15a-5p has been shown to inhibit cell growth and induce tumor cell apoptosis, primarily through the downregulation of Bcl-2 and Bcl-xl [57].

A correlation has been identified between the aberrant expression of miR-16-5p and the counts of lymphocytes and platelets, as well as the concentrations of D-dimer and ferritin. Notably, reduced levels of miR-16-5p (with a fold change of 0.72) were observed in patients categorized as critically ill. Moreover, miR-16-5p is implicated in the synthesis of cytokines and chemokines [18]. miR-16 has been reported to exert significant inhibitory effects on cell proliferation and invasion, promoting cell apoptosis and suppressing cell cycle progression [58]. Specifically, miR-16 directly targets PDCD4, leading to the suppression of inflammatory macrophage activation in atherosclerosis through pathways such as MAPK and NF-κB, as well as downstream inflammatory cytokines [59]. There is also evidence to suggest that miR-16 suppresses the secretion and mRNA expression of pro-inflammatory factors like IL-6 and TNF-α. Conversely, it enhances the secretion and mRNA expression of the anti-inflammatory factor IL-10 [59]. In the context of insulin sensitivity, miR-16 enhances this sensitivity by attenuating macrophage-mediated pro-inflammatory responses, including those related to TNF-α, IL-6, and IFN-β [60]. Furthermore, miR-16 potentially shifts macrophage polarization from M2 to M1 phenotypes and activates CD4+T cells by downregulating PD-L1 [61]. In terms of disease-specific expression, miR-16 is upregulated in conditions such as RA, JIA, IBD, and pSS, whereas it is found to be downregulated in the serum of patients with AS and SLE [62]. The expression pattern of miR-15a-5p and miR-16-5p in pregnant women post-COVID-19 vaccination has yet to be elucidated, but its augmented expression might also be related to the potential ability of these two to restore T-cell immunity and clear infected cells.

Recent studies have highlighted a correlation between miR-223-3p and fibrosis development in patients exhibiting persistent hyper-reactivity to inflammatory stimuli. Inhibition of miRNA-223-3p displayed an upregulation of anti-inflammatory factors and a consequent reduction in pulmonary inflammation [63]. These findings underscore the proactive role of miRNA-223 in modulating pulmonary inflammatory responses triggered by SARS-CoV-2 [64,65]. miR-223-3p has been shown to directly inhibit the expression of the S protein and replication of SARS-CoV-2 [15]. Intriguingly, serum exosomes from younger individuals have the capability to suppress SARS-CoV-2 replication and S protein expression, yet this inhibitory effect appears to be markedly diminished in elderly and diabetic patients [15]. MiR-223 serves as a crucial anti-inflammatory miRNA, modulating immune responses by activating immune cells such as macrophages, neutrophils, and dendritic cells [66]. It is essential in managing reactions to infectious diseases like viral hepatitis, HIV-1, Helicobacter pylori infection, and sepsis. MiR-223 strikingly balances protective immune responses against potential host damage from excessive inflammation [66]. This equilibrium stems from its ability to regulate the function of macrophages and neutrophils and control inflammasome activation under pathological scenarios. Moreover, in pregnant women post-COVID-19 vaccination, a decrease in the expression of miR-223-3p was observed with an increase in vaccine doses. Although the exact cause remains elusive, it might be related to its reduced expression being anti-inflammatory and its involvement in modulating immune responses.

It is known that with an increase in vaccine doses, the body’s Nab values and Nab inhibition increase, and the protection against different variants of SARS-CoV-2 is also enhanced. Explaining vaccine efficacy from the perspective of miRNA, we can infer that, as the vaccine boosts maternal and fetal antibodies and protection, corresponding physiological changes occur in the body. Since many miRNA expressions might interact with cytokines related to immunity or inflammatory reactions, corresponding changes would also be observed. Our research reflects the variations in miRNA expression in maternal blood and neonatal umbilical cord blood as the vaccination doses increase. Although many mechanisms remain unclear, based on the increased or decreased expression of miRNA, we can speculate that after vaccination, miRNA may be involved in modulating IL-6R, T-cell immunity, enhancing protection against organ damage, and the potential ability to clear infected cells.

To the best of our knowledge, this is the first study examining miRNA expression in peripheral maternal blood and neonatal umbilical cord blood of pregnant women vaccinated with different doses of COVID-19 vaccines. The primary limitation of our study was the small sample size. However, it is challenging to collect samples from pregnant women who have not been vaccinated or have only received two doses of the vaccine, making it difficult to further increase our sample numbers. Another limitation of our pilot study is the constrained sample size, which restricts our capacity for further comparative analysis, such as assessing variations due to differences in vaccine administration timing. This aspect remains challenging to evaluate within the scope of our research. Naturally, our study also lacks validation. Therefore, it is difficult in our present study to elucidate the impact of differences in vaccination timing and related details. To gain a more comprehensive understanding of miRNA, we can further investigate the expression of downstream proteins, or collect more samples to detect the miRNAs that are found with significantly different expressions in our present study. This is something we can focus on in our future research.

## 5. Conclusions

From our pilot study, it can be observed that changes in miRNA may reflect the immune response-related molecular mechanism in the mother and fetus as the vaccine dose rises. This is associated with the elevated expression of miR-451a, miR-1972, miR-486-5p, miR-423-5p, miR-15a-5p, and miR-16-5p, as well as the decreased expression of miR-223-3p. The shifts in miRNA provide us with deeper insights into the vaccine’s mechanism on pregnant women. In the future, further validation can be pursued via examination of downstream protein expressions or additional target miRNAs.

## Figures and Tables

**Table 1:** List of participants in our study.

Case	Age	Dose 1	Dose 2	Dose 3	Dose 4	Tdap	Flu	BMI	Baby BW (gm)	Baby Gender
1	33	Moderna (GA 2 weeks)	Moderna (GA 38 weeks)	NA	NA	Yes	Yes	26.56	3070	Male
2	33	Moderna (GA 26 weeks)	Moderna (GA 36 weeks)	NA	NA	No	No	28.0	3130	Male
3	27	Moderna (GA 15 weeks)	Moderna (GA 19 weeks)	Moderna (GA 34 weeks)	NA	Yes	Yes	26.55	2880	Female
4	38	Moderna (8 weeks before preg)	Moderna (GA 15 weeks)	Moderna (GA 34 weeks)	NA	No	No	23.30	3045	Male
5	38	BNT (25 weeks before preg)	BNT (20 weeks before preg)	BNT (8 weeks before preg)	Bi-valent Moderna BA.1 (GA 36 weeks)	Yes	Yes	28.12	2520	Male

Tdap—tetanus toxoid, reduced diphtheria toxoid, and acellular pertussis vaccines; flu—influenza vaccine; BMI—body mass index; BW—body weight; Moderna—Spikevax (elasomeran) COVID-19 vaccine (previously called the mRNA-1273 Moderna vaccine); BNT—Pfizer BioNTech (BNT162b2) COVID-19 vaccine; Bi-valent Moderna BA.1—Moderna COVID-19 bivalent (SPIKEVAX Bivalent Original/Omicron BA.1) vaccine; GA—gestational age; and preg—pregnancy; NA—not available.

**Table 2:** The intersection of maternal blood and cord blood miRNA with significant expression between different dosages of COVID-19 vaccines.

The Intersection of Maternal Blood and Neonatal Cord Blood Samples	Maternal (ΔΔCq)	Neonatal (ΔΔCq)
Difference between 2 and 3 doses of vaccine		
hsa-miR-451a	−4.69	−1.60
hsa-miR-1972	−6.41	−1.00
Difference between 2 and 4 doses of vaccine		
hsa-miR-451a	−4.14	−2.16
hsa-miR-486-5p	−0.62	−2.93
hsa-miR-1972	−9.39	−4.66
Difference between 3 and 4 doses of vaccine		
hsa-miR-423-5p	−2.10	−3.40
hsa-miR-1972	−2.98	−3.66

**Table 3:** miRNA with significant differential expression between “2 dose/3 dose” and “2 dose/4 dose”.

	2 Dose vs. 3 Dose (ΔΔCq)	2 Dose vs. 4 Dose (ΔΔCq)
hsa-miR-15a-5p	0.69	−1.83
hsa-miR-16-5p	−0.38	−1.84
hsa-miR-223-3p	1.90	0.07
hsa-miR-423-5p	−1.23	−3.58

**Table 4:** The pathway functions of miR-451a, miR-1972, and miR-423-5p.

ID	Description	*p* Value	Gene
miR-451a Pathway Function			
hsa04151	PI3K-Akt signaling pathway	0.0000045	AKT1/BCL2/MYC/IL6R/IKBKB/ATF2/TSC1/YWHAZ
hsa04915	Estrogen signaling pathway	0.0000415	AKT1/MMP2/MMP9/BCL2/ATF2
hsa01522	Endocrine resistance	0.00017	AKT1/MMP2/MMP9/BCL2
hsa04668	TNF signaling pathway	0.000285	AKT1/MMP9/IKBKB/ATF2
hsa04152	AMPK signaling pathway	0.000383	CAB39/AKT1/RAB14/TSC1
hsa04926	Relaxin signaling pathway	0.000489	AKT1/MMP2/MMP9/ATF2
hsa04150	mTOR signaling pathway	0.000999	CAB39/AKT1/IKBKB/TSC1
hsa04218	Cellular senescence	0.000999	AKT1/MYC/ETS1/TSC1
hsa04630	JAK-STAT signaling pathway	0.00115	AKT1/BCL2/MYC/IL6R
hsa01521	EGFR tyrosine kinase inhibitor resistance	0.00151	AKT1/BCL2/IL6R
miR-1972 Pathway Function			
hsa03020	RNA polymerase	0.007212425	POLR1B/POLR1E
hsa04722	Neurotrophin signaling pathway	0.009993483	CALM3/TP53/NGFR
hsa00600	Sphingolipid metabolism	0.016944596	PSAPL1/SPTLC3
hsa04218	Cellular senescence	0.020619785	CALM3/TP53/CDK6
hsa01524	Platinum drug resistance	0.030841833	TP53/GSTO2
hsa04115	p53 signaling pathway	0.030841833	TP53/CDK6
miR-423-5p Pathway Function			
hsa04927	Cortisol synthesis and secretion	0.0000820	PLCB1/SP1/KCNK3/CREB1/ADCY3
hsa04928	Parathyroid hormone synthesis, secretion, and action	0.0000866	PLCB1/SP1/ARRB2/CREB1/MMP17/ADCY3
hsa04915	Estrogen signaling pathway	0.000367	PLCB1/TFF1/CALM3/SP1/CREB1/ADCY3
hsa04713	Circadian entrainment	0.00054	NOS1AP/PLCB1/CALM3/CREB1/ADCY3
hsa04925	Aldosterone synthesis and secretion	0.000566	PLCB1/CALM3/KCNK3/CREB1/ADCY3
hsa04916	Melanogenesis	0.005216	PLCB1/CALM3/CREB1/ADCY3
hsa04922	Glucagon signaling pathway	0.006393	PGAM4/PLCB1/CALM3/CREB1
hsa04935	Growth hormone synthesis, secretion, and action	0.009522	PLCB1/STAT5B/CREB1/ADCY3
hsa04062	Chemokine signaling pathway	0.010493	PLCB1/ARRB2/STAT5B/CXCL12/ADCY3
hsa04110	Cell cycle	0.011559	E2F3/ABL1/YWHAE/MCM7

## Data Availability

Data is contained within the article or supplementary material.

## Supplementary Materials

The following supporting information can be downloaded at: https://www.mdpi.com/article/10.3390/vaccines11121814/s1, Figure S1. Principal Component Analysis of the miRNA data. Two_maternal_396 as case 1 maternal; two_fetal_396 as case 1 neonatal; two_maternal_877 as case 2 maternal; two_fetal_877 as case 2 neonatal; three_maternal_765 as case 3 maternal; three_fetal_765 as case 3 neonatal; three_maternal_358 as case 4 maternal; three_fetal_358 as case 4 neonatal; four_maternal_334 as case 5 maternal; and four_fetal_334 as case 5 neonatal. Figure S2. The intersection of maternal blood and cord blood miRNAs with significant expression between those receiving two and three doses of COVID-19 vaccines. Fetal_UP, upregulation in neonatal samples; fetal_DOWN, downregulation in neonatal samples; mom_UP, upregulation in maternal samples; and mom_DOWN, downregulation in maternal samples. Figure S3. The intersection of maternal blood and cord blood miRNAs with significant expression between those receiving two and four doses of COVID-19 vaccines. Fetal_UP, upregulation in neonatal samples; fetal_DOWN, downregulation in neonatal samples; mom_UP, upregulation in maternal samples; and mom_DOWN, downregulation in maternal samples. Figure S4. The intersection of maternal blood and cord blood miRNAs with significant expression between those receiving three and four doses of COVID-19 vaccines. Fetal_UP, upregulation in neonatal samples; fetal_DOWN, downregulation in neonatal samples; mom_UP, upregulation in maternal samples; and mom_DOWN, downregulation in maternal samples. Table S1. Nab inhibition rate to Omicron BA.5 SARS-CoV-2 from both maternal blood and neonatal cord blood. NA, not available due to insufficient sample amount for laboratory study; Nab, neutralizing antibody. Table S2. The pathway function of miR-486-5p. Table S3. The pathway function of miR-16-5p. Table S4. The pathway function of miR-15a-5p. Table S5. The pathway function of miR-223-3p.
