# Supplementary material for: COVID-19 Vaccination in Pregnancy: Pilot Study for Maternal and Neonatal MicroRNA Profiles
Source: Vaccines (Basel). 2023 Dec 4;11(12):1814. doi: 10.3390/vaccines11121814 (PMC10747030; doi:10.3390/vaccines11121814)
Supplement: Supplementary file 1 [file vaccines-11-01814-s001.zip › Supplementary table.pdf]

Table S1. Nab inhibition rate to Omicron BA.5 SARS-CoV-2 from both maternal blood and neonatal cord blood

Case	Vaccine doses	Maternal blood Nab inhibition rates (BA.5)	Neonatal cord blood Nab inhibition rates (BA.5)
1	2	NA	5.56%
2	2	NA	8.74%
3	3	14.18%	5.48%
4	3	35.69%	23.74%
5	4	85.31%	NA

NA, not available due to insufficient sample amount for laboratory study; Nab, neutralizing antibody

Table S2. The pathway function of miR-486-5p

ID	Description	p value	gene
hsa04218	Cellular senescence	0.001815	SERPINE1 / CDK4 / PTEN / FOXO1
hsa04115	p53 signaling pathway	0.001896	SERPINE1 / CDK4 / PTEN
hsa04350	TGF-beta signaling pathway	0.003895	ID4 / FBN1 / SMAD1
hsa00640	Propanoate metabolism	0.005246	PCCA / EHHADH
hsa04068	FoxO signaling pathway	0.00978	IGF1R / PTEN / FOXO1
hsa04910	Insulin signaling pathway	0.011046	SOCS2 / FOXO1 / CBL
hsa00280	Valine, leucine, and isoleucine degradation	0.011536	PCCA / EHHADH
hsa04550	Signaling pathways regulating pluripotency of stem cells	0.012403	ID4 / IGF1R / SMAD1
hsa04213	Longevity regulating pathway - multiple species	0.018779	IGF1R / FOXO1
hsa01521	EGFR tyrosine kinase inhibitor resistance	0.029527	IGF1R / PTEN

Table S3. The pathway function of miR-16-5p

ID	Description	pvalue	gene
hsa04151	PI3K-Akt signaling pathway	0.000000000925	MCL1 / CDK6 / CCND3 / CCND1 / CCNE1 / PPP2R5C / LAMC1 / ITGA2 / FGF2 / EIF4E / EGFR / BCL2 / RPS6 / BRCA1 / AKT3 / CCND2 / VEGFA / HSP90B1 / MYB / CHUK / TP53 / KDR / FGFR1 / GRB2 / RPS6KB1 / MTOR / EIF4B / CCNE2 / GNB1 / YWHAH / IGF1R / RAF1 / KRAS / CDKN1A / PIK3R1 / BDNF / FGFR4 / YWHAQ / PHLPP2 / PRKAA1 / GNG12
hsa04150	mTOR signaling pathway	0.00000000327	LAMTOR5 / EIF4E / RPS6 / AKT3 / WNT3A / LAMTOR3 / LAMTOR2 / CHUK / GRB2 / RPS6KB1 / FLCN / MTOR / RPS6KA3 / EIF4B / SLC7A5 / IGF1R / RAF1 / KRAS / FZD6 / STRADB / PIK3R1 / RICTOR / WNT4 / PRKAA1 / FZD9
hsa04110	Cell cycle	0.0000000350	CDK1 / CDC14B / CDK6 / CCND3 / CCND1 / CCNE1 / PLK1 / CCND2 / ANAPC16 / TP53 / CDC25A / ANAPC13 / CDC27 / CCNE2 / WEE1 / YWHAH / E2F3 / CDKN1A / CHEK1 / ORC4 / YWHAQ
hsa01521	EGFR tyrosine kinase inhibitor resistance	0.000000526	FGF2 / EIF4E / EGFR / BCL2 / RPS6 / AKT3 / VEGFA / KDR / GRB2 / RPS6KB1 / MTOR / IGF1R / RAF1 / KRAS / PIK3R1
hsa01522	Endocrine resistance	0.00000184	JUN / CCND1 / NOTCH2 / EGFR / BCL2 / AKT3 / TP53 / GRB2 / RPS6KB1 / MTOR / E2F3 / IGF1R / RAF1 / KRAS / CDKN1A / PIK3R1
hsa04218	Cellular senescence	0.00000485	CDK1 / CDK6 / CCND3 / CCND1 / CCNE1 / SQSTM1 / AKT3 / CCND2 / MCU / TP53 / CDC25A / MTOR / CCNE2 / E2F3 / BTRC / RAF1 / KRAS / CDKN1A / CHEK1 / PIK3R1
hsa04115	p53 signaling pathway	0.00000643	CDK1 / CDK6 / CCND3 / CCND1 / CCNE1 / BCL2 / CCND2 / PPM1D / TP53 / CCNE2 / CDKN1A / CHEK1 / ZMAT3
hsa04510	Focal adhesion	0.00000670	JUN / CCND3 / CCND1 / LAMC1 / ITGA2 / EGFR / BCL2 / AKT3 / CCND2 / VEGFA / ZYX / KDR / GRB2 / ACTG1 / CRKL / VCL / IGF1R / RAF1 / PAK2 / PIK3R1 / CRK / VAV2 / TLN1

hsa04012	ErbB signaling pathway	0.00000735	JUN / EGFR / AKT3 / GRB2 / RPS6KB1 / ABL2 / MTOR / CRKL / RAF1 / KRAS / PAK2 / CDKN1A / PIK3R1 / CRK
hsa04010	MAPK signaling pathway	0.00000839	JUN / HSPA1A / HSPA1B / FGF2 / EGFR / CACNA2D1 / AKT3 / VEGFA / LAMTOR3 / CHUK / TP53 / KDR / FGFR1 / GRB2 / RPS6KA3 / CRKL / MAP3K7 / IGF1R / RAF1 / KRAS / PAK2 / PPM1A / TAOK1 / ELK4 / BDNF / FGFR4 / MAP4K2 / CRK / GNG12

Table S4. The pathway function of miR-15a-5p

ID	Description	pvalue	gene
hsa04151	PI3K-Akt signaling pathway	0.0000000328	MYB / CCND2 / BCL2 / MCL1 / CCND1 / CCNE1 / BRCA1 / AKT3 / HSP90B1 / VEGFA / NFKB1 / CHUK / TP53 / FGF7 / RPS6KB1 / YWHAH / FGF2 / PPP2R5C / ITGA2 / PIK3R1 / PHLPP1 / FGFR4 / LAMC1 / GNB1 / CDK6 / CCNE2 / YWHAQ / PHLPP2 / GRB2 / PRKAA1 / GNG12
hsa04110	Cell cycle	0.000000762	CDC25A / CCND2 / CDC14B / CCND1 / CCNE1 / ANAPC16 / TP53 / YWHAH / WEE1 / CHEK1 / CDK6 / ORC4 / CDK1 / CCNE2 / ANAPC13 / YWHAQ
hsa04115	p53 signaling pathway	0.0000458	CCND2 / BCL2 / CCND1 / CCNE1 / TP53 / CHEK1 / CDK6 / ZMAT3 / CDK1 / CCNE2
hsa04550	Signaling pathways regulating pluripotency of stem cells	0.0000693	BMI1 / WNT3A / AKT3 / KLF4 / FGF2 / FZD6 / PIK3R1 / FGFR4 / AXIN2 / JARID2 / GRB2 / FZD9 / ACVR2A / RIF1
hsa04010	MAPK signaling pathway	0.00013	HSPA1A / JUN / AKT3 / VEGFA / NFKB1 / CHUK / TP53 / FGF7 / CRKL / HSPA1B / PAK2 / PPM1A / TAOK1 / FGF2 / ELK4 / FGFR4 / MAP4K2 / GRB2 / CRK / MAP3K7 / GNG12
hsa04910	Insulin signaling pathway	0.000173	PHKB / AKT3 / CRKL / FASN / FOXO1 / RPS6KB1 / PIK3R1 / PRKAR2A / PHKA1 / GRB2 / CRK / PRKAA1 / FLOT2
hsa04218	Cellular senescence	0.000177	CDC25A / CCND2 / CCND1 / CCNE1 / AKT3 / NFKB1 / TP53 / FOXO1 / BTRC / CHEK1 / PIK3R1 / CDK6 / CDK1 / CCNE2
hsa04660	T-cell receptor signaling pathway	0.000211	JUN / AKT3 / NFKB1 / CHUK / IFNG / PAK2 / PIK3R1 / PDCD1 / GRB2 / VAV2 / MAP3K7
hsa04310	Wnt signaling pathway	0.000432	WNT3A / CCND2 / JUN / CCND1 / TP53 / BTRC / ZNRF3 / FZD6 / CSNK1E / PRICKLE2 / AXIN2 / MAP3K7 / FZD9 / CCDC88C
hsa01522	Endocrine resistance	0.000546	BCL2 / JUN / CCND1 / AKT3 / TP53 / CARM1 / RPS6KB1 / PIK3R1 / GRB2 / NOTCH2

Table S5. The pathway function of miR-223-3p

ID	Description	pvalue	gene
hsa04218	Cellular senescence	0.00000000515	E2F1 / FOXO1 / CDK2 / ATM / FOXO3 / IL6 / TP53 / RRAS2 / MDM2 / PTEN / CDK4 / CDK6
hsa04068	FoxO signaling pathway	0.00000000952	CHUK / IGF1R / SLC2A4 / FOXO1 / CDK2 / ATM / FOXO3 / IL6 / MDM2 / S1PR1 / PTEN
hsa04115	p53 signaling pathway	0.000000146	CDK2 / ATM / TP53 / SESN3 / MDM2 / PTEN / CDK4 / CDK6
hsa04110	Cell cycle	0.0000101	E2F1 / CDK2 / ATM / CDC27 / TP53 / MDM2 / CDK4 / CDK6
hsa01522	Endocrine resistance	0.0000166	E2F1 / IGF1R / CARM1 / SP1 / TP53 / MDM2 / CDK4
hsa04151	PI3K-Akt signaling pathway	0.0000335	CHUK / IGF1R / HSP90B1 / CDK2 / FOXO3 / IL6 / TP53 / MDM2 / ITGB1 / PTEN / CDK4 / CDK6
hsa04621	NOD-like receptor signaling pathway	0.000144	CHUK / NAMPT / IL6 / CXCL2 / TRPV2 / TXNIP / NLRP3 / STAT1
hsa04625	C-type lectin receptor signaling pathway	0.000227	CHUK / IL6 / RRAS2 / MDM2 / NLRP3 / STAT1
hsa04137	Mitophagy - animal	0.000331	E2F1 / FOXO3 / SP1 / TP53 / RRAS2
hsa04211	Longevity regulating pathway	0.00088	IGF1R / FOXO1 / FOXO3 / TP53 / SESN3
