# Supplementary figures and images for: COVID-19 Vaccination in Pregnancy: Pilot Study for Maternal and Neonatal MicroRNA Profiles
Source: Vaccines (Basel). 2023 Dec 4;11(12):1814. doi: 10.3390/vaccines11121814 (PMC10747030; doi:10.3390/vaccines11121814)

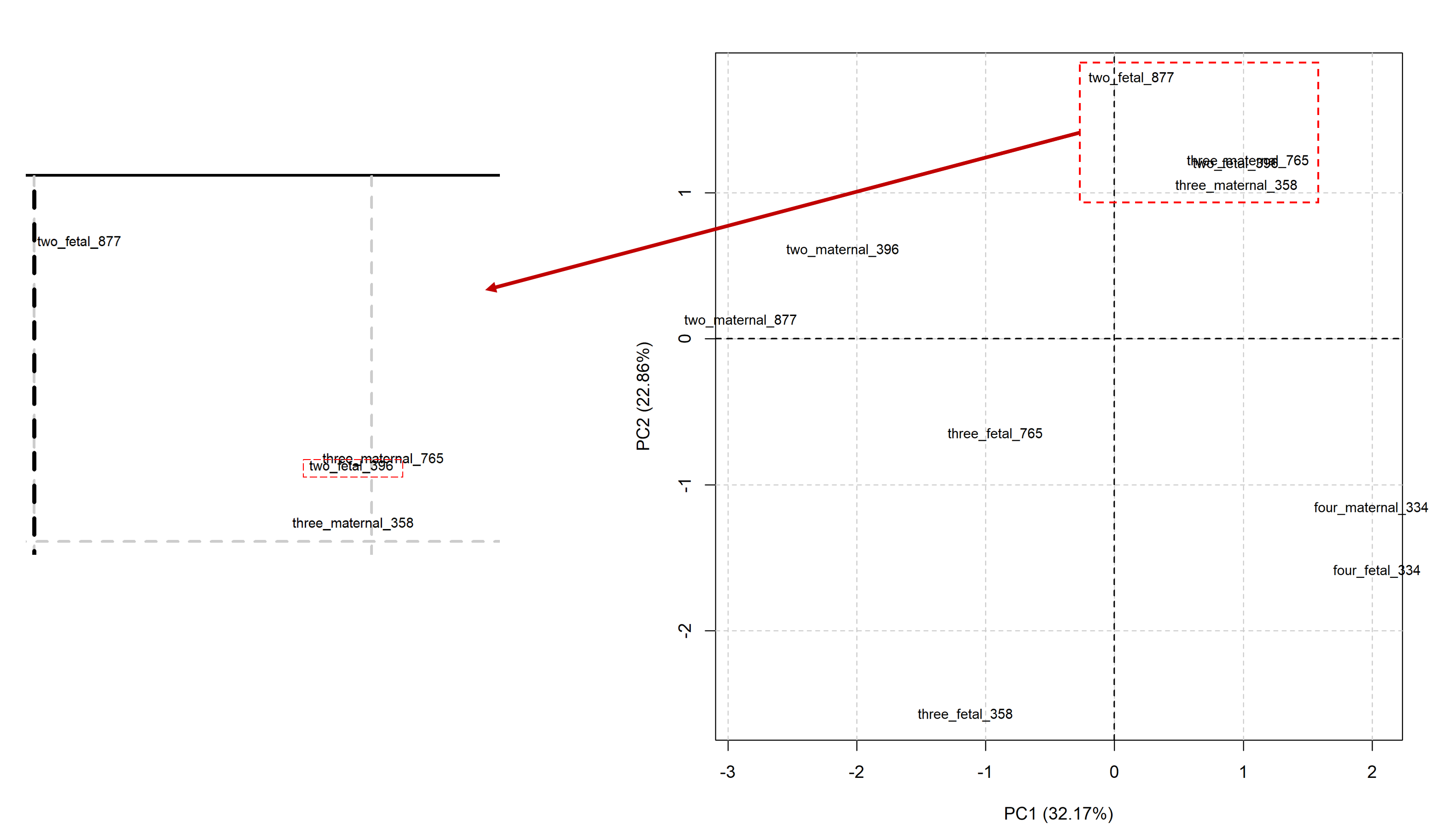

Supplement: Supplementary file 1 [file vaccines-11-01814-s001.zip › Fig. S1-20231009.tif]

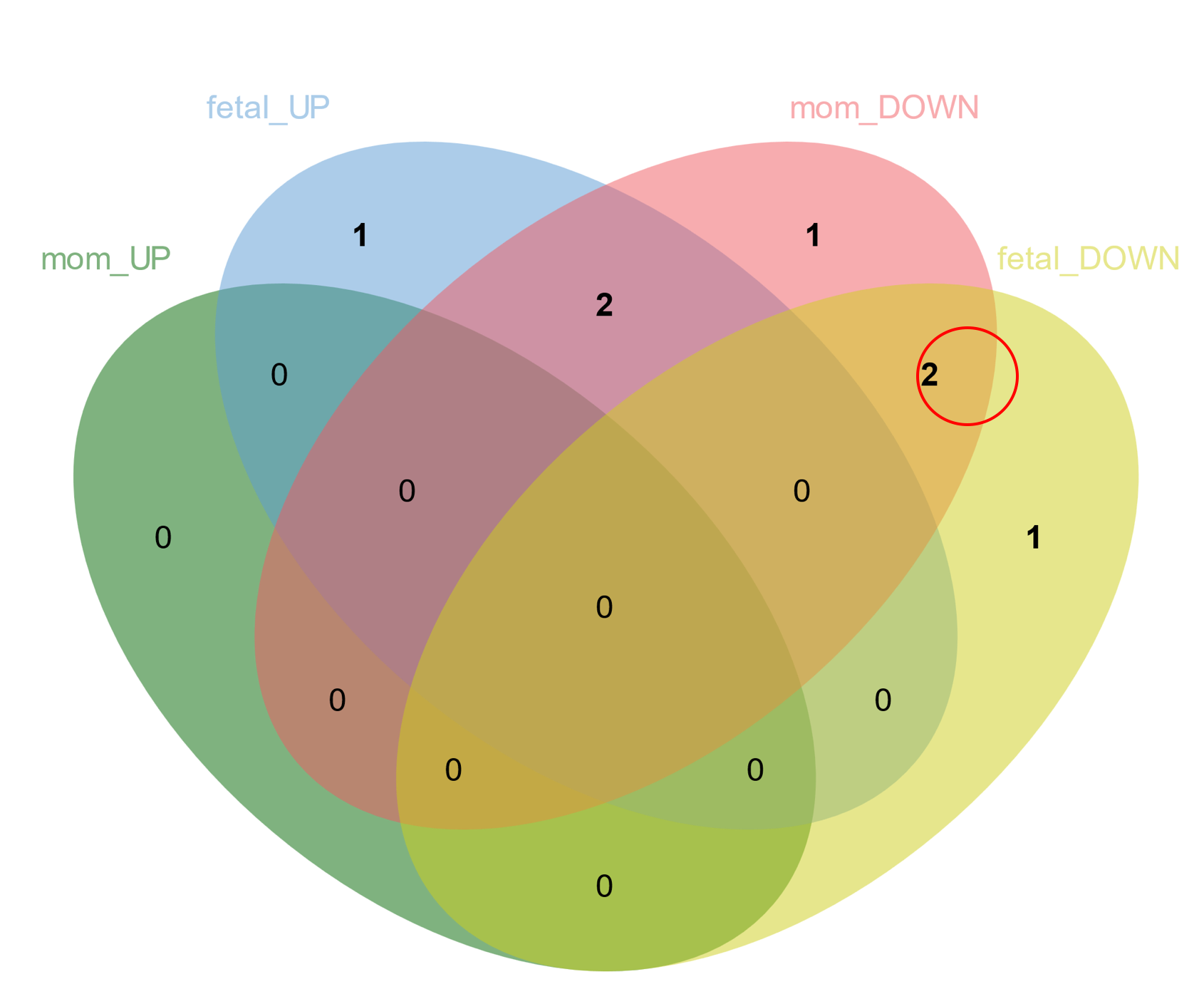

Supplement: Supplementary file 1 [file vaccines-11-01814-s001.zip › Fig. S2-20231009.tif]

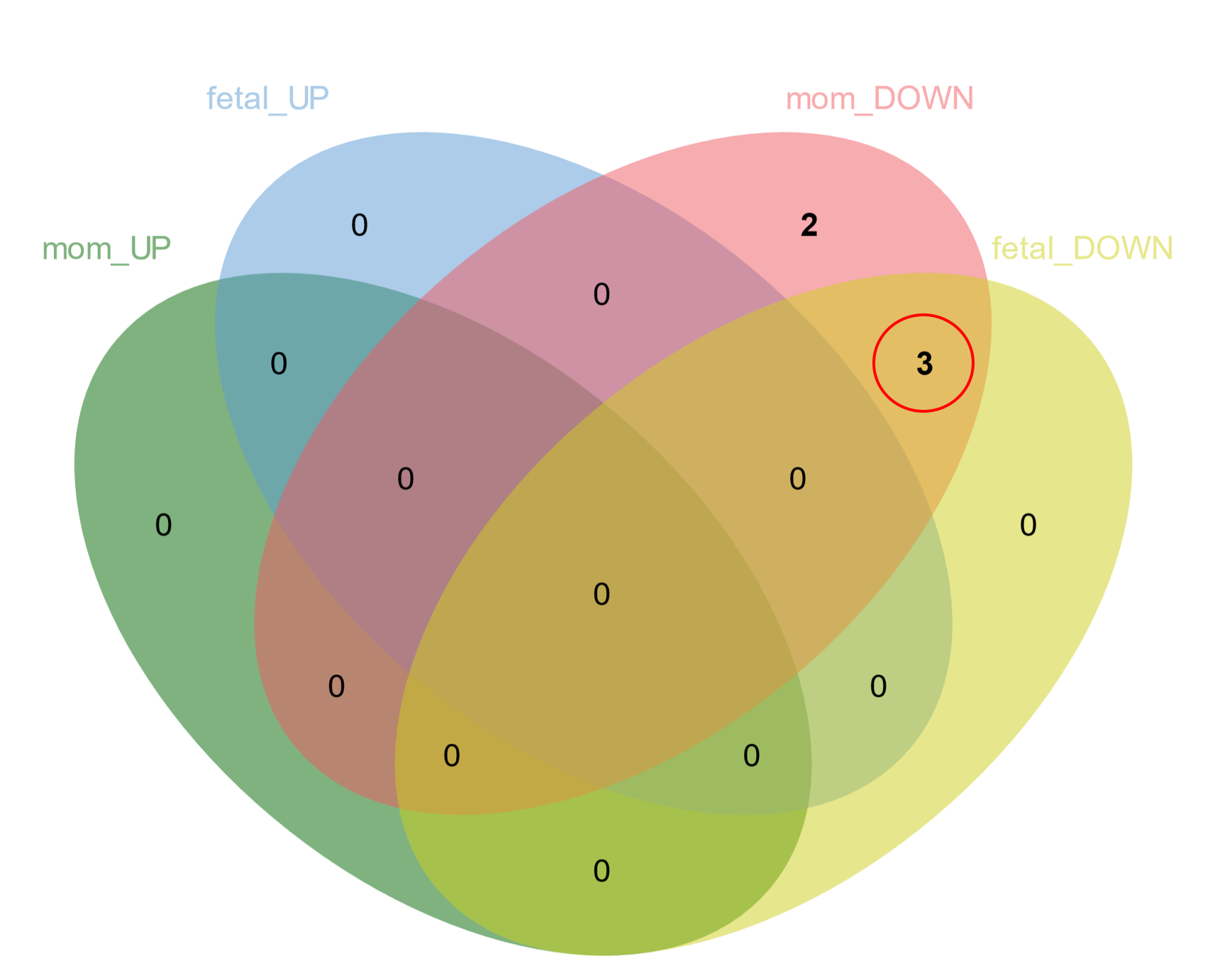

Supplement: Supplementary file 1 [file vaccines-11-01814-s001.zip › Fig. S3-20231009.tif]

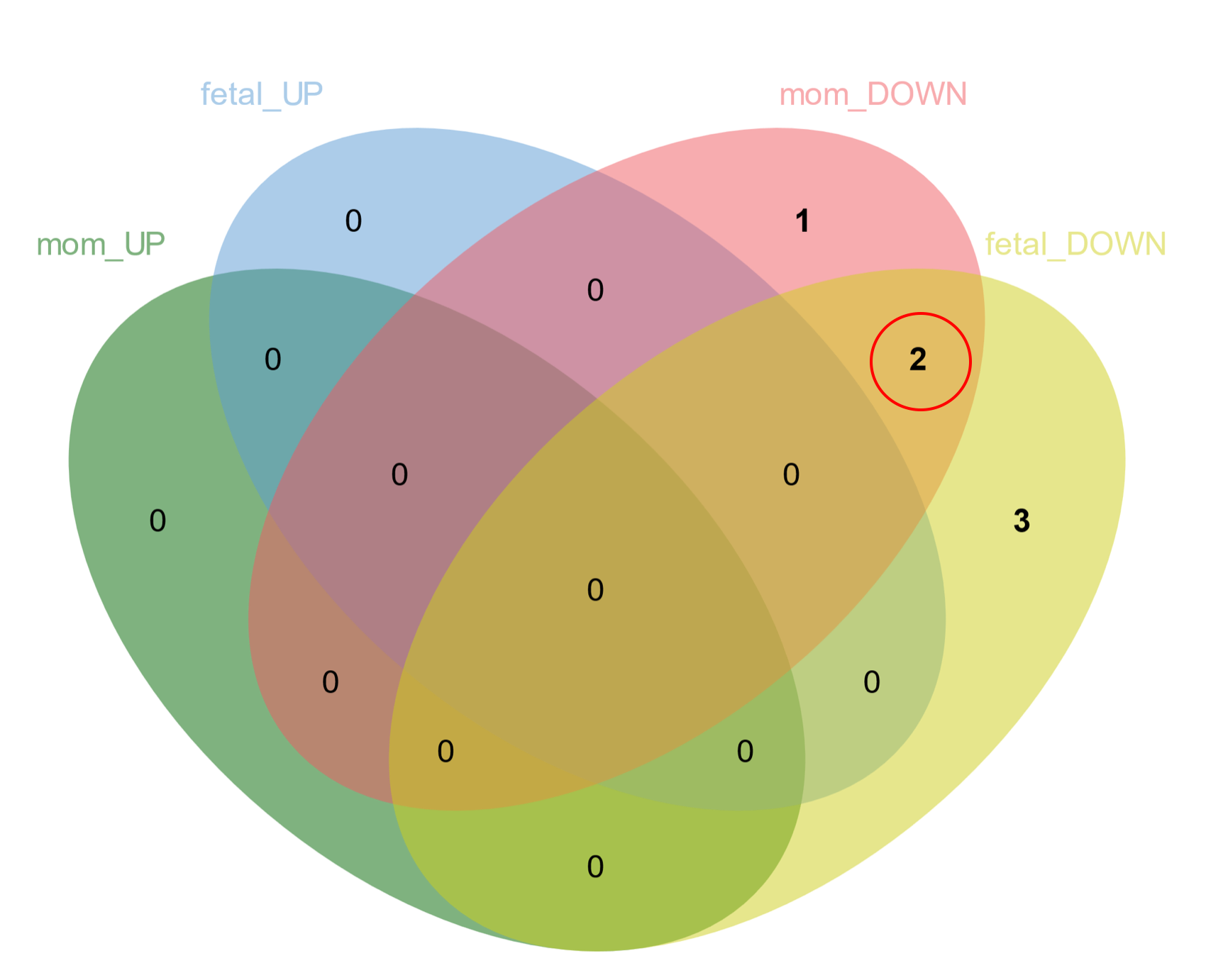

Supplement: Supplementary file 1 [file vaccines-11-01814-s001.zip › Fig. S4-20231009.tif]
